# Synthesis of a dehydrodieugenol B derivative as a lead compound for visceral leishmaniasis—mechanism of action and *in vivo* pharmacokinetic studies

**DOI:** 10.1128/aac.00831-24

**Published:** 2024-10-09

**Authors:** Maiara Amaral, Maiara M. Romanelli, Hannah Asiki, Joana Bicker, Daniela P. Lage, Camila S. Freitas, Noemi N. Taniwaki, Joao Henrique G. Lago, Eduardo A. F. Coelho, Amílcar Falcão, Ana Fortuna, Edward A. Anderson, Andre G. Tempone

**Affiliations:** 1 Laboratory of Physiopathology, Instituto Butantan, São Paulo, Brazil; 2 Instituto de Medicina Tropical, Faculdade de Medicina, Universidade de Sao Paulo, Sao Paulo, Brazil; 3 Department of Chemistry, Chemistry Research Laboratory, University of Oxford, Oxford, United Kingdom; 4 Laboratory of Pharmacology and Pharmaceutical Care, Faculty of Pharmacy, University of Coimbra, Coimbra, Portugal; 5 Coimbra Institute for Biomedical Imaging and Translational Research (CIBIT), Institute of Nuclear Sciences Applied to Health (ICNAS), University of Coimbra, Coimbra, Portugal; 6 Laboratório de Pesquisa do Programa de Pós-Graduação em Ciências da Saúde: Infectologia e Medicina Tropical, Faculdade de Medicina, Universidade Federal de Minas Gerais, Belo Horizonte, Minas Gerais, Brazil; 7 Laboratory of Electron Microscopy, Instituto Adolfo Lutz, São Paulo, Brazil; 8 Center for Natural and Human Sciences, Federal University of ABC, Santo Andre, Brazil; 9 Departamento de Patologia Clínica, COLTEC, Universidade Federal de Minas Gerais, Belo Horizonte, Minas Gerais, Brazil; The Children's Hospital of Philadelphia, Philadelphia, Pennsylvania, USA

**Keywords:** *Leishmania*, natural product derivative, mechanism of action, pharmacokinetic

## Abstract

Leishmaniasis is a parasitic neglected tropical disease, affecting 12 million people. Available treatments present several limitations, with an increasing number of resistance cases. In the search for new chemotherapies, the natural product dehydrodieugenol B was used as a scaffold for the synthesis of a series of derivatives, resulting in the discovery of the promising analog [4-(4-(5-allyl-3-methoxy-2-((4-methoxybenzyl)oxy)phenoxy)-3-methoxybenzyl)morpholine, **1**]. In this work, we investigated the effect of compound **1** on cell signaling in *Leishmania (L.) infantum*, culminating in cell death, as well as its immunomodulatory effect in the host cell. Additionally, we performed a pharmacokinetic profile study in an animal model. After treatment, compound **1** induced the alkalinization of acidocalcisomes and concomitant Ca^2+^ release in the parasite. These events may induce depolarization of the mitochondrial potential, with successive collapse of the bioenergetic system, leading to a reduction of ATP and reactive oxygen species (ROS) levels. The analysis of total proteins and protein profile by matrix-assisted laser desorption ionization–time of flight mass spectrometry (MALDI-TOF/MS) demonstrated that compound **1** also altered the parasite proteins after treatment. Transmission electron microscopy studies revealed ultrastructural damage to mitochondria; together, these data suggest that compound **1** may promote autophagic cell death. Additionally, compound **1** also induced an immunomodulatory effect in host cells, with a reduction of Th1 and Th2 cytokine response, characterizing an anti-inflammatory compound. The obtained pharmacokinetic profile in rats enhances the potential of the compound, with a mean plasma half-life (T_1/2_) of 21 h. These data reinforce the potential of compound **1** as a new lead for future efficacy studies.

## INTRODUCTION

Neglected tropical diseases cause about 17% of the world’s deaths and generate a huge economic and social impact (1). Among them, leishmaniasis has a high morbidity and mortality, affecting more than 12 million people worldwide. Visceral leishmaniasis (VL) is the most severe clinical form, which is predominantly caused by two species: *Leishmania* (*L*.) *donovani* and *Leishmania* (*L*.) *infantum*. VL is endemic mainly to low-income populations, with 90% of cases concentrated in India, China, Nepal, Bangladesh, Kenya, Somalia, Ethiopia, Sudan, South Sudan, and Brazil. The disease is fatal if untreated, causing mortality rates of 10% to 20%, with the number of deaths varying from 20,000 to 50,000 people per year (2, 3). Despite this scenario, VL continues to receive low investment from the pharmaceutical industry. The disease has a limited therapeutic arsenal composed mainly of three drugs: pentavalent antimonials, amphotericin B, and miltefosine. These treatments present several limitations, including elevated toxicity, parenteral administration (except miltefosine), hospitalization (except miltefosine), long dosage regimens, and the high cost of the whole treatment. Thus, the development of new therapies for this disease is an urgent goal (4–8).

**Fig 1:**
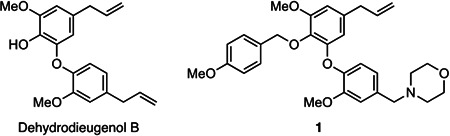
Structure of the natural product dehydrodieugenol B and its synthetic derivative, compound **1**.

In the search for new drug leads for visceral leishmaniasis, our group reported the anti-*Leishmania* activity of neolignans isolated from the Brazilian plant *Nectandra leucantha* (Lauraceae) (9, 10). Among them, dehydrodieugenol B (Fig. 1) was one of the most selective and, in a subsequent analysis, demonstrated a promising potential as a prototype for new derivatives with anti-*L*. (*L*.) *infantum* activity (11, 12). To facilitate more diverse structural scaffold modifications, a total synthesis of this neolignan was developed, which enabled structure-activity relationship studies (SAR) (13). Further synthetic and biological studies identified [4-(4-(5-allyl-3-methoxy-2-((4-methoxybenzyl)oxy)phenoxy)-3-methoxybenzyl)morpholine] (**1**, Fig. 1) as showing the most encouraging results, with an IC_50_ value of 9.7 µM against amastigotes of *L*. (*L*.) *infantum* and a SI > 20 (12). Additionally, compound **1** fulfilled the criteria suggested by the DND*i* for new hit compounds (5). In this work, we investigated the impact of compound **1** on the cell signaling of *L. (L.) infantum,* culminating in cell death. Additionally, we performed a pharmacokinetic profile study in an animal model aiming toward a future *in vivo* efficacy study.

## MATERIALS AND METHODS

### General experimental procedures

Sytox Green, MTT, H_2_DCFDA, JC-1, ATP Determination Kit, propidium iodide, acridine orange, fluo-4 AM, and BCA Protein Assay Kit were purchased from Molecular Probes (Invitrogen). Fetal bovine serum (FBS) was obtained from Gibco and e QIAamp DNA Mini Kit from Qiagen. The α-cyano-4-hydroxy-cinnamic acid matrix (HCCA) and the bacterial standard of *Escherichia coli* DH5-alpha protein extract (BTS) were purchased from Bruker-Daltonics. The BD OptEIA Mouse ELISA set kits for IL-4, IL-10, IL-12, and INF-γ were obtained from BD Biosciences. All other reagents not mentioned were purchased from Sigma-Aldrich (Merck). Luminescence, absorbance, and fluorescence readings were performed using FilterMax F5 Multi-Mode Microplate Reader spectrofluorometer (Molecular Devices) or Atunne NxT flow cytometer (Thermo Fisher Scientific). High-performance liquid chromatography (HPLC) analyses were performed on the Nexera-i LC-2040C equipment (Shimadzu) and mass spectrometry on the MALDI-TOF/MS BrukerAutoflex III (Bruker-Daltonics).

### Animals

Golden hamsters (*Mesocricetus auratus*) and BALB/c mice were obtained from the animal breeding facility of the Instituto Adolfo Lutz—SP. All procedures were previously approved by the Animal Use Ethics Committee (CEUA IAL 05/2018 and IMT-USP 000404A). Healthy adult Wistar rats were purchased from certified animal suppliers (Charles River Laboratory, L'Arbresle, France) and maintained under controlled environmental conditions with *ad libitum* access to tap water and food. The experimental procedures were conducted in accordance with the European Directive (2010/63/EU) on the protection of laboratory animals and the Portuguese animal welfare law (Decree-Law 113/2013). They were also reviewed and approved by the Portuguese National Authority for Animal Health, Phytosanitation and Food Safety (DGAV—Direção Geral de Alimentação e Veterinária) and Animal Welfare and Ethics Body (ORBEA—Órgão Responsável pelo Bem dos Animais).

### Parasites and mammalian cell maintenance

*L*. (*L*.) *infantum* (MHOM/BR/1972/LD) promastigotes were maintained in M199 medium supplemented with 10% FBS and 0.25% hemin, pH 7.2 at 24°C. Amastigotes were isolated from golden hamsters (*M. auratus*) by differential centrifugation of infected spleens and the parasite’s number was determined according to Stauber (14). Peritoneal macrophages were obtained from the peritoneal cavity of BALB/c mice and maintained in RPMI-1640 medium supplemented with 10% FBS, pH 7.2 at 37°C.

### Preparation of compound 1

Compound **1** [4-(4-(5-allyl-3-methoxy-2-((4-methoxybenzyl)oxy)phenoxy)-3-methoxybenzyl)morpholine] was prepared as described previously (12). **1** displayed nuclear magnetic resonance (NMR), electrospray ionization high-resolution mass spectrometry (ESI-HRMS), and infrared (IR) data identical to those previously reported.

### Hemolytic activity

Erythrocytes collected from BALB/c mice were added to a U-shaped bottom 96-wells microplate at 3% (vol/vol) in phosphate-buffered saline (PBS), treated with compound **1** serially diluted (6.5 to 200 µM) in PBS and incubated for 2 h at 24°C. To determine the hemolytic activity, cell supernatants were collected and analyzed in a spectrophotometer (570 nm) (15). Bidistilled water was employed as a positive control and untreated erythrocytes as a negative control.

### Mechanism of action

The IC_50_ of compound **1** against intracellular amastigotes of *L. (L.) infantum* was previously determined and resulted in a value of 9.7 ± 2.0 µM after 24 h incubation using 1 × 10^6^ promastigotes/well (12). All experiments were performed with *L*. (*L*.) *infantum* promastigotes in the late-grown phase using a Hanks-Balanced Salt Solution medium supplemented with D-glucose (10 mM), exceptions were described. To establish the ideal time and concentration to investigate the mechanism of action (MoA), a new IC_50_ assay was performed in promastigotes in short-time incubation. Parasites (2 × 10^6^/well) were treated with compound **1** serially diluted (1.6 to 300 µM) for 1, 2, 3, and 4 h, at 24°C. Parasite viability was determined using the MTT colorimetric method (16). Untreated cells were employed as a negative control.

#### Plasma membrane permeability

Promastigotes (2 × 10^6^/well) were pretreated with Sytox Green (1 µM) for 15 min, at 24°C. Then, compound **1** (169.2 µM) was added and the fluorescence was monitored every 20 min up to 4 h in a spectrofluorometer, with excitation and emission wavelengths of 485 and 535 nm, respectively (17). Triton X-100 (0.5% vol/vol) was employed as positive control and untreated parasites as negative control (18).

#### Mitochondrial membrane electric potential (ΔΨ_m_)

Promastigotes (2 × 10^6^/well) were treated with compound **1** (169.2 µM) for 2 and 4 h, at 24°C. Then, JC-1 (10 µM) was added and the parasites were incubated for 20 min. Fluorescence was measured in a flow cytometer with an excitation wavelength of 488 nm and emission of 530 (BL-1) and 574 nm (BL-2). Mitochondrial membrane potential was established through the ratio of BL-2/BL-1 channels (19). Carbonyl cyanide m-chlorophenylhydrazone (CCCP) (10 µM) was employed as positive control and untreated parasites as negative control.

#### Adenosine triphosphate

Promastigotes (2 × 10^6^/well) were treated with compound **1** (169.2 µM) for 2 and 4 h, at 24°C. Subsequently, the parasites were lysed with Triton X-100 (0.5%) and mixed with ATP Determination Kit standard reaction buffer (1 mM DTT, 0.5 mM luciferin, and 1.25 µg/mL luciferase), according to the manufacturer’s instructions. The samples were observed in a luminometer and an ATP curve (1 to 6,000 nM) was used as a standard for quantification (20). CCCP (10 µM) was employed as positive control and untreated parasites as negative control.

#### Reactive oxygen species

Promastigotes (2 × 10^6^/well) were treated with compound **1** (169.2 µM) for 2 and 4 h, at 24°C. Later, H_2_DCFDA (5 µM) was added and the parasites were incubated for 15 min. Fluorescence was monitored in a spectrofluorometer with excitation and emission wavelengths of 485 and 535 nm, respectively (19). Sodium azide (10 mM) was employed as positive control and untreated parasites as negative control.

#### Intracellular Ca^2+^

Promastigotes (2 × 10^6^/well) were pretreated with Fluo-4 AM (5 µM) in PBS, for 1 h at 24°C. Then, parasites were treated with compound **1** (169.2 µM). Fluorescence was measured every 20 min up to 4 h in a spectrofluorometer, with excitation and emission wavelengths of 485 nm and 535 nm, respectively (21). Triton X-100 0.5% (vol/vol) was employed as positive control and untreated parasites as negative control.

#### Acidocalcisomes

Promastigotes (2 × 10^6^/well) were pretreated with acridine orange (4 µM) in PBS, for 5 min at 24°C. The parasites were treated with compound **1** (169.2 µM). Fluorescence was monitored every 20 min up to 4 h in a spectrofluorometer, with excitation and emission wavelengths of 485 and 535 nm, respectively (22). Nigericin (4 µM) was employed as positive control and untreated parasites as negative control (23).

#### Cell cycle

Promastigotes (2 × 10^6^/well) in exponential phase, were treated with compound **1** (169.2 µM) in M199 medium supplemented with 10% FBS and 0.25% hemin, pH 7.2, for 24 h at 24°C. In sequence, 70% (vol/vol) ice-cold ethanol was used for cell permeabilization overnight at −20°C. Then, the parasites were incubated with propidium iodide (10 µg/mL) and RNase A (20 µg/mL) for 30 min. Fluorescence was observed in the flow cytometer with excitation and emission wavelengths of 488 nm and 574 nm, respectively (24). Miltefosine (25 µM) was employed as positive control and untreated parasites as negative control (25).

#### DNA content

Promastigotes (1 × 10^7^/well) were treated with compound **1** (169.2 μM) in M199 medium supplemented with 10% FBS and 0.25% hemin, pH 7.2, for 24 h at 24°C. Then, the genetic material was extracted using the QIAamp DNA Mini Kit, according to the manufacturer’s instructions. Total DNA quantification was performed in a NanoDrop ND-1000 spectrophotometer (260/280 nm). The purified genetic material (300 ng) was analyzed by electrophoresis for 1 h at 60 mV in a 2% agarose gel with ethidium bromide (0.5 µg/mL). Visualization was performed under UV light in a MiniBIS Pro transilluminator (DNR Bio Imaging System) (21). H_2_O_2_ (10 mM) was employed as positive control and untreated parasites as negative control (26).

#### Ultrastructural studies

Promastigotes (3 × 10^7^ parasites/well) were treated with compound **1** (169.2 µM) for 2, 4, and 6 h at 24°C. Subsequently, parasites were fixed in glutaraldehyde solution (2.5% wt/vol) and post-fixed in osmium tetroxide (1% wt/vol). The samples were gradually dehydrated in acetone and embedded in Epon 812 resin. Ultrathin sections were stained with uranyl acetate and lead citrate (27). The material was analyzed under a transmission electron microscope (JEOL JEM-1011). Untreated parasites were employed as a negative control.

### Immunomodulatory response

#### Nitric oxide

Peritoneal macrophages (5 × 10^5^/well) were infected with *L*. (*L*.) *infantum* amastigotes (10:1 amastigotes/macrophage) for 24 h, at 37°C with 5% CO_2_. Later, the cells were treated with compound **1** serially diluted (7.5 to 60 µM), under the same conditions described above. After 48 h, the supernatants were collected and incubated with Griess reagent (28). The samples were observed in a spectrophotometer (570 nm) and a NaNO_2_ curve (0 to 400 µM) was used as a standard for quantification. LPS (25 µg/mL) was employed as the positive control and untreated cells as the negative control (29).

#### Cytokines

Peritoneal macrophages (1 × 10^6^/well) were infected with *L*. (*L*.) *infantum* promastigotes (10:1 amastigotes/macrophage) for 24 h, at 37°C with 5% CO_2_. Subsequently, compound **1** (10 and 60 µM) was added and the incubation followed the same conditions described above. Cell supernatants were collected after 48 and 72 h and the cytokines (IL-4, IL-10, IL-12, and INF-γ) were measured using the BD OptEIA Mouse ELISA set kits, according to the manufacturer’s instructions. Concanavalin A (5 µg/mL) was employed as positive control and untreated cells as negative control.

### HPLC quantification of compound 1 in biological samples

The detection and quantification of compound **1** in the blood matrices were performed after sample processing, using a liquid-liquid extraction methodology. First, 100 µL of plasma was enriched with 10 µL of PI (0.8 µg/mL). Subsequently, 500 µL of hexane was added and the mixture was vortexed for 1 min and centrifuged at 22,082 × *g* for 5 min. The organic phase was collected in glass tubes and the aqueous phase was subjected to the liquid-liquid extraction procedure again. In the end, the combined organic samples were completely evaporated at 60°C under nitrogen flow and reconstituted in 100 µL of 40:60 ACN:H_2_O + 0.1% orthophosphoric acid (pH 2.08). Finally, the extracts were centrifuged at 22,082 × *g* for 2 min and injected into the chromatographic system. Chromatographic analyses by HPLC were performed using a C_18_ column (Purospher Star LiChroCART, 55 × 4 mm, 3 µm) with gradient elution (Table 1).

**TABLE 1:** Chromatographic and validation conditions for HPLC quantification of compound **1** in plasma^*a*^

Parameter	Value
Mobile phase	A: H_2_O + orthophosphoric acid 0.1% (pH 2.08) B: ACN [30% (0–1 min); 30% to 65% (1–2.5 min); 65% (2.5–5.0 min); 65% to 30% (5.0–7.0 min); 30% (7.0–10.0 min)]
Flow (mL/min)	1.0
Temperature (°C)	25
Wavelength (nm)	220
Injection volume (μL)	20
Running time (min)	10
Calibration range (μg/mL)	0.03–6
LLOQ (μg/mL)	0.03
Regression equation	y = 0.9249x − 0.0086
Determination coefficient (r^2^)	0.9991
Interday precision (% CV)	≤5.0
Interday accuracy (% BIAS)	−5.12 – 3.61

^*a*^ ACN, acetonitrile; LLOQ, lower limit of quantification; %BIAS, deviation from nominal concentration; %CV, coefficient of variation.

Dehydrodieugenol B (0.8 µg/mL) was used as the internal standard (PI). Method validation was performed following the guidelines for bioanalytical methods validation of the Food and Drug Administration and European Medicines Agency (29, 30). Thus, the acceptance criteria set out in these guides were adopted for the parameters of selectivity, linearity, precision, accuracy, lower limit of quantification (LLOQ), and stability. Quality controls of the lower limit of quantification (QCLOQ—0.03 µg/mL), low (QC1—0.08 µg/mL), medium (QC2—2 µg/mL), and high (QC3—5 µg/mL), were also established in accordance with the guides. All analytical conditions and validation parameters are shown in Table 1, demonstrating the method’s accuracy, precision, and robustness. In addition, stability was established after exposure to different conditions, mimicking storage and handling throughout the experimental process (Table 2).

**TABLE 2:** Stability of compound **1** in plasma, under different handling and storage conditions

Condition	Stability/reference analyte concentration (%)	
	0.08 µg/mL	5.00 µg/mL
Processed sample		
2 h at room temperature	98.35	100.43
24 h at 4°C	99.12	98.84
Unprocessed sample		
2 h at room temperature	86.54	90.90
6 h at 4°C	91.74	95.92
24 h at 4°C	87.07	91.65
15 days at −20°C	85.36	90.53
30 days at −20°C	92.66	93.24
Freezing/thawing	88.58	97.49

### *In vivo* pharmacokinetic study

Compound **1** (5 mg/kg) was administered (10 mL/kg in saline-sodium-chloride-0.9%) by intraperitoneal route, to six Wistar rats. Blood samples were collected from the caudal vein at 30 min, 1, 2, 3, 5, 6, 8, 12, and 24 h post-dosing. Plasma samples were prepared as described in the previous section and analyzed by HPLC. Concentrations below the LLOQ were considered as zero. Pharmacokinetic profile was obtained using the WinNonlin 6.4 software, determining different parameters, including half-life time (T_½_); maximum time (T_max_); maximum plasma concentration (C_max_); area under the curve from time zero to the last measurable concentration time (AUC_0-t_); area under the curve from zero to infinity (AUC_0-inf_); percentage of area under the curve extrapolated from the last measurable concentration time to infinity (AUC_extrap_); and meantime of residence (MRT).

#### Statistical analysis

The determination of IC_50_ values was obtained using sigmoid dose-response curves, while the statistical significance between samples was assessed using the *P* values by the one-way analysis of variance method using Tukey’s Multiple Comparison test. All analyses were evaluated using the Graph Pad Prism 5 software. The samples were assessed in duplicate and the assays were reproduced at least twice, with a representative experiment presented.

## RESULTS

### Hemolytic activity

After 2 h of treatment with compound **1,** no hemolysis could be observed in the tested concentrations (6.25 to 200 µM) (data not shown). The incubation with bidistilled water resulted in 100% of hemolysis.

### Mechanism of action studies

**Fig 2:**
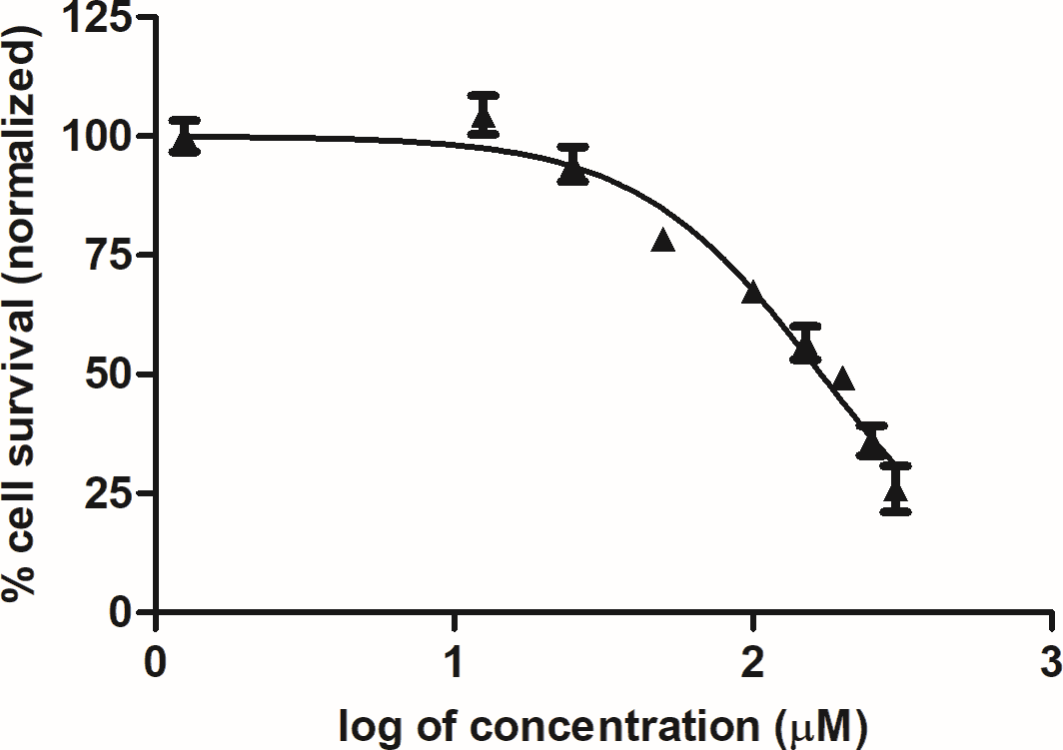
Determination of the 50% inhibitory concentration of compound **1** for MoA studies using short-time incubation. The promastigotes (2 × 10^6^/well) were treated for 4 h and the viability of cells was determined by the MTT assay at 570 nm.

To determine the adequate concentration of compound **1** for the MoA studies, we performed a short-time incubation assay to determine the 50% inhibitory concentration. The results showed an IC_50_ value of 169.2 µM after 4 h of treatment (Fig. 2). Therefore, all MoA studies presented below, were carried out using this concentration (exceptions were described).

#### Plasma membrane permeability

**Fig 3:**
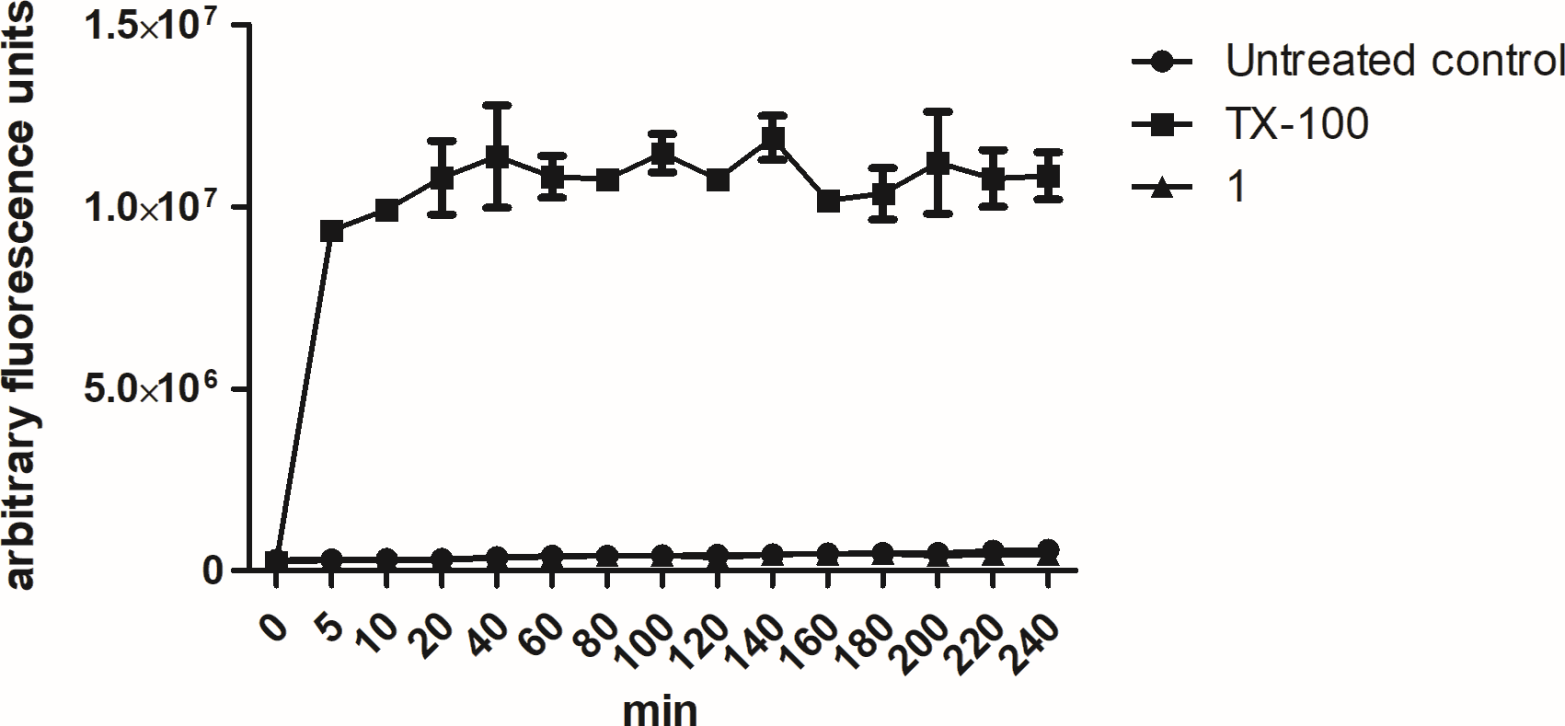
Evaluation of plasma membrane permeability in *L*. (*L*.) *infantum* promastigotes treated with compound **1** (169.2 µM), measured in a spectrofluorometer for 4 h, using Sytox Green dye (excitation 485 nm and emission 535 nm). Untreated and Triton X-100 (0.5%) treated parasites were used as negative and positive controls, respectively. Fluorescence was reported as a percentage of Triton X-100 in 240 min (100%). **P < 0.0055 in relation to the control.

The plasma membrane permeability was evaluated by spectrofluorometric analysis using the fluorescent probe SYTOX Green. As presented in Fig. 3, treatment with compound **1** (169.2 µM) showed no alteration in the plasma membrane permeability of *Leishmania*, with fluorescence levels similar to untreated parasites, even after 4 h of incubation. Triton X-100 was used as a positive control, promoting significative permeabilization of the plasma membrane.

#### Mitochondrial membrane electric potential (ΔΨ_m_)

**Fig 4:**
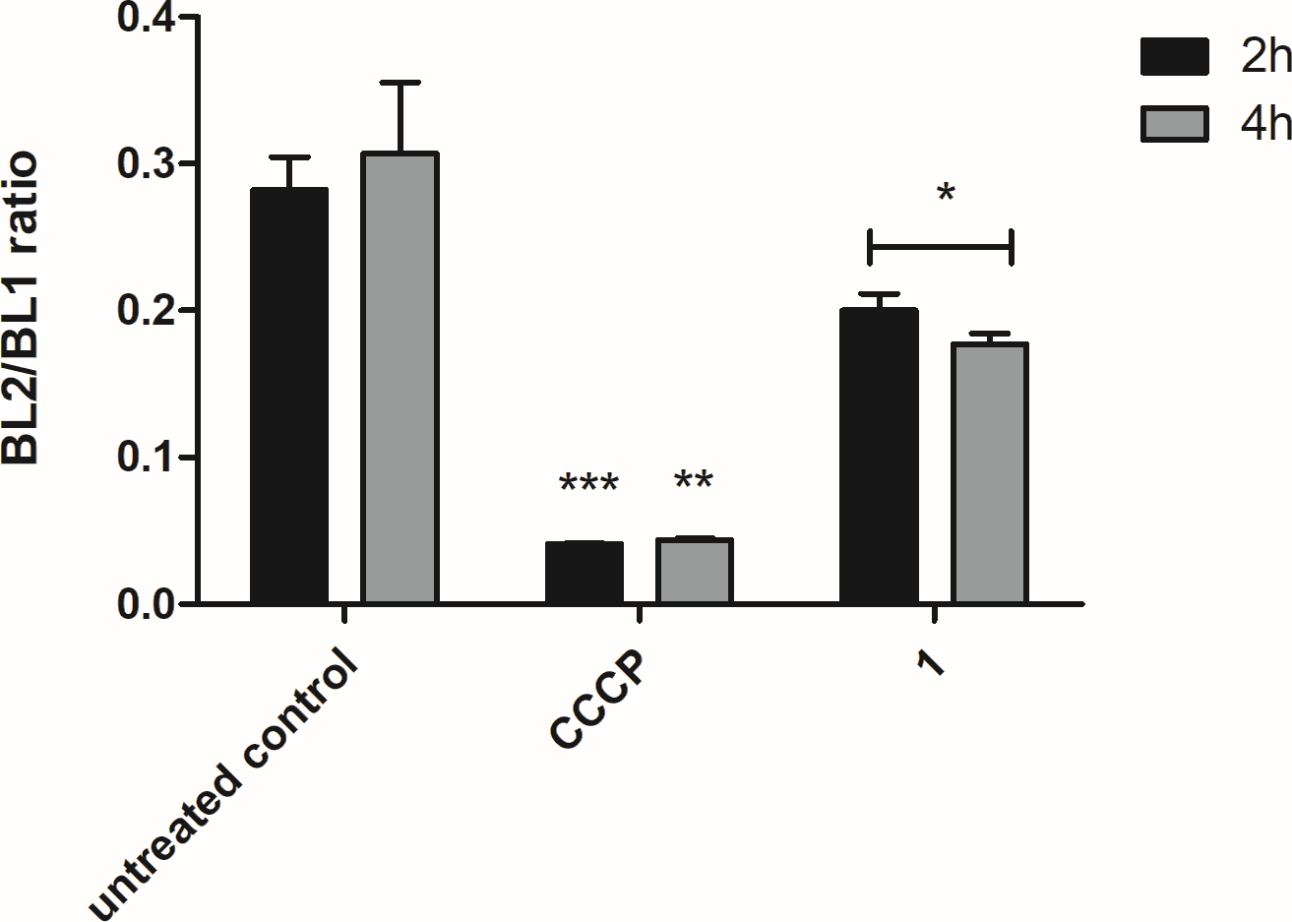
Evaluation of mitochondrial membrane potential in *L*. (*L*.) *infantum* promastigotes, after treatment with compound **1** (169.2 µM) for 2 and 4 h, measured by flow cytometry using JC-1 dye (excitation 488 nm and emission 530/574 nm). Untreated and CCCP-treated (10 µM) parasites were used as negative and positive controls, respectively. Fluorescence was reported as the ratio between BL-2 (574 nm) and BL-1 (530 nm). ***P < 0.0001, **0.006 and *0.05 in relation to control.

The mitochondrion was subsequently investigated for possible damage, using flow cytometry analysis with the fluorescent probe JC-1. After incubation with compound **1,** a significant depolarization (*P* < 0.05) of the membrane electric potential was observed when compared to untreated parasites, with an extended effect up to 4 h. Maximum depolarization was obtained using the positive control CCCP (Fig. 4).

#### Adenosine triphosphate

**Fig 5:**
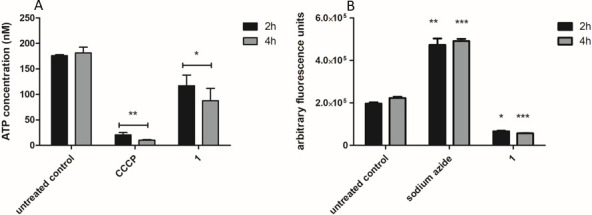
Evaluation of adenosine triphosphate and reactive oxygen species levels in *L*. (*L*.) *infantum* promastigotes after treatment with compound **1** (169.2 µM) for 2 and 4 h. (**A**) Monitored in a spectroluminometer using the ATP Determination Kit. Untreated and CCCP (10 µM) treated parasites were used as negative and positive control, respectively. (**B**) Measured in a spectrofluorometer using the probe H_2_DCFDA (excitation 485 nm and emission 535 nm). Untreated and sodium azide (10 mM) treated parasites were used as negative and positive controls, respectively. Fluorescence is reported as a percentage of untreated parasites (100%). **P value 0.0012 and *P value 0.05 in relation to the control.

Due to the intense alteration of the mitochondria of promastigotes, the ATP levels were investigated using a bioluminescence assay with recombinant firefly luciferase and its substrate D-luciferin. As illustrated in Fig. 5A, treatment with compound **1** (169.2 µM) resulted in a significant reduction in ATP concentration (*P* < 0.05) at both times, when compared to untreated parasites. The positive control CCCP (10 µM) was used for ATP levels to decrease.

#### Reactive oxygen species

Considering the mitochondrial damages observed in the promastigotes after treatment with compound **1**, the reactive oxygen species (ROS) content was investigated using a spectrofluorometer and the probe H_2_DCFDA. According to the obtained data (Fig. 5B), compound **1** (169.2 µM) induced a significant reduction of the ROS levels when compared to untreated parasites (*P* < 0.05 in 2 h and 0.0001 in 4 h). In contrast, the positive control (10 mM sodium azide) increased ROS production.

#### Intracellular Ca^2+^

**Fig 6:**
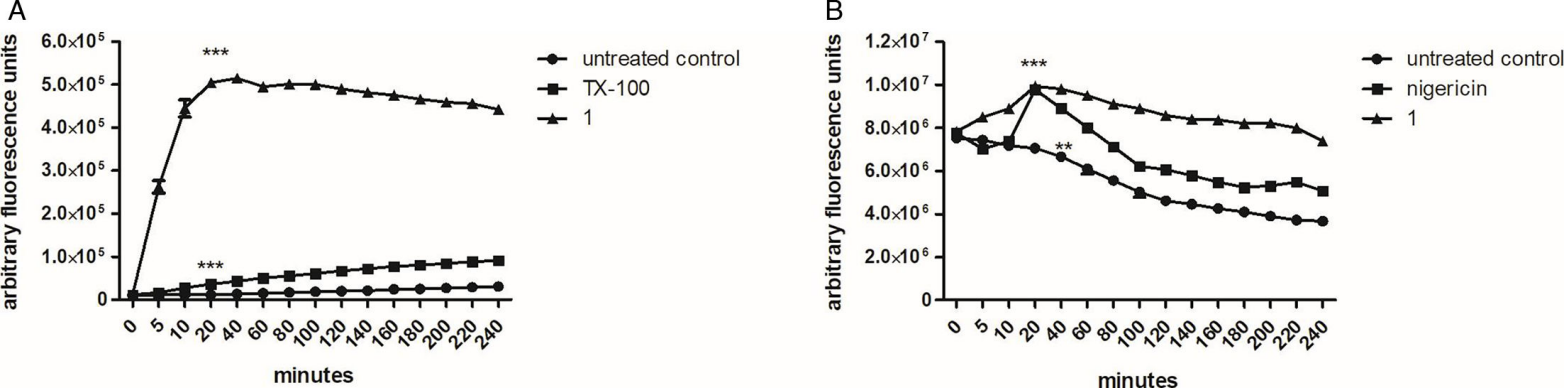
Evaluation of intracellular Ca^2+^ levels and acidocalcisomes alkalinization in *L*. (*L*.) *infantum* promastigotes after treatment with compound **1** (169.2 µM) for 4 h. (**A**) Monitored in a spectrofluorometer using the Fluo-4 AM dye (excitation 485 nm and emission 535 nm). Untreated and Triton X100 (0.5%) treated parasites were used as negative and positive controls, respectively. Fluorescence was reported as a percentage of untreated parasites in 0 min (100%). (**B**) Measured in a spectrofluorometer using the fluorophore acridine orange (excitation 485 nm and emission 535 nm). Untreated and nigericin (4 µM) treated parasites were used as negative and positive controls, respectively. Fluorescence was reported as a percentage of nigericin in 20 min (100%). ***P < 0.0003 and **0.0025 in relation to the control.

The intracellular content of Ca^2+^ was investigated in promastigotes using a spectrofluorometer and the probe Fluo-4 AM. According to the data presented in Fig. 6A, treatment with compound **1** (169.2 µM) induced a fast and increased Ca^2+^ level, with statistical differences (*P* < 0.0001) observed within the first time point (20 min), when compared to the untreated group (control). This difference remained constant through the entire incubation period of 4 h and the Ca^2+^ up-regulation was superior to that observed with the positive control, Triton X-100.

#### Acidocalcisomes

Considering the intense cellular alterations caused by compound **1** in the Ca^2+^ levels of promastigotes, the integrity of the acidocalcisomes was investigated using a spectrofluorometer and the probe acridine orange. As demonstrated in Fig. 6B, after 20 min of treatment with compound **1** (169.2 µM) the fluorescence levels were significantly higher than those of the untreated parasites (*P* < 0.0003), demonstrating an interference of the compound in this organelle. A similar effect was observed in the positive control, the K+/H+ exchanger, nigericin.

#### Cell cycle

**Fig 7:**
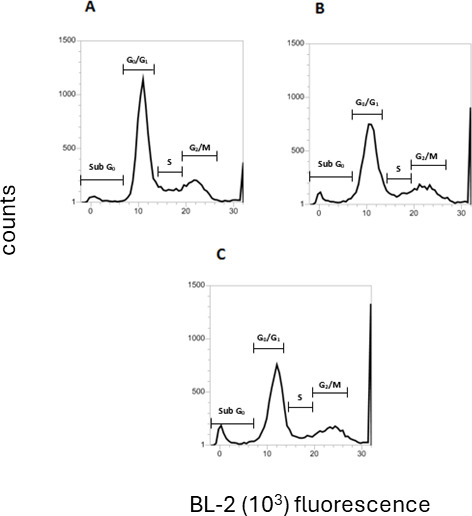
Cell cycle evaluation of *L*. (*L*.) *infantum* promastigotes after treatment for 24 h, measured by flow cytometry using propidium iodide (excitation 488 nm and emission 574 nm). (**A**) Untreated control, (**B**) miltefosine, and (**C**) compound **1** (169.2 µM). Sub G_0_: fragmented DNA, cell death; G_0_/G_1_: diploid cells (2N); S: DNA replication; G_2_/M: cells with duplicated DNA.

Given the alterations observed in the metabolism of the parasite after treatment with compound **1,** we investigated the cellular division of the parasite using flow cytometry (Fig. 7; Table 3). Treatment with compound **1** (169.2 µM) for 24 h induced a significant increase in the Sub G_0_ population (*P* < 0.05) when compared to untreated parasites. An interference in the G_0_/G_1_ phase was also observed, with a significant decrease in the cell number (*P* < 0.0001). No considerable changes were verified in the S and G_2_/M phases. The positive control, miltefosine, demonstrated a similar effect to the studied compound.

**TABLE 3:** Cell cycle alterations of *L*. (*L*.) *infantum* promastigotes after treatment with compound **1** for 24 h^*a*^

Group	Proportion of parasites in different phases of the cell cycle (% ± SD)			
	Sub G_0_	G_0_/G_1_	S	M/G_2_
Untreated control	3.4 ± 0.4	61.3 ± 0.3	12.3 ± 0.2	18.2 ± 0.0
**1**	7.5 ± 0.7*	46.6 ± 0.7***	11.1 ± 1.3	17.9 ± 0.5
Miltefosine	7.1 ± 0.6*	53.8 ± 0.1***	10.5 ± 0.3	18.1 ± 0.4

^*a*^ Sub G_0_: fragmented DNA, cell death; G_0_/G_1_: diploid cells (2N); S: DNA replication; G_2_/M: cells with duplicated DNA; SD: standard deviation. ***P < 0.0001 and *P < 0.05 in relation to the control.

#### DNA content

**Fig 8:**
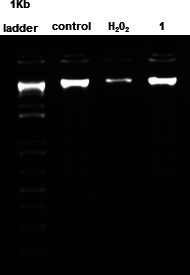
Evaluation of DNA integrity in *L*. (*L*.) *infantum* promastigotes after treatment with compound **1** (169.2 µM) for 24 h, using 2% agarose gel electrophoresis with ethidium bromide (0.5 µg/mL). Untreated and H_2_O_2_ (10 mM) treated parasites were used as negative and positive controls, respectively. A ladder of 1 Kb was used as a standard.

In accordance with the previous assay to evaluate the cell division of the parasite, the DNA content of promastigotes was also investigated after treatment with compound **1**. As shown in Fig. 8, no alterations in the integrity of the genetic content were observed, with a band of similar intensity and characteristics to the untreated parasites. The positive control, H_2_O_2_ (10 mM), exhibited a lower intensity band, illustrating DNA fragmentation.

#### Ultrastructure studies

**Fig 9:**
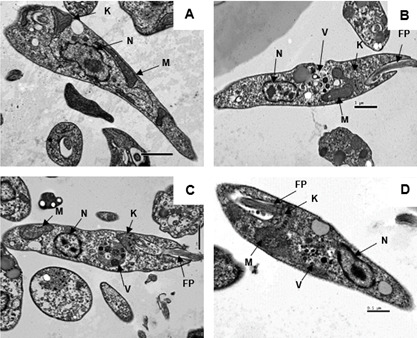
Evaluation of ultrastructural alterations in *L*. (*L*.) *infantum* promastigotes, using transmission electron microscopy. (**A**) Untreated control; treatment with compound **1** (169.2 µM) for (**B**) 2 h, (**C**) 4 h, and (**D**) 6 h. N, nucleus; K, kinetoplast; M, mitochondria; FP, flagellar pocket; and V, vacuole.

As illustrated in Fig. 9A, untreated parasites exhibited a standard morphology with intact membranes and unharmed organelles. After 2 h of treatment with compound **1** (169.2 µM), an increased volume of the mitochondria was observed (Fig. 9B). These effects intensified with prolonged exposure to compound **1** resulting in losses of mitochondrial cristae at later times of incubation (Fig. 9C and D). Additionally, autophagic vacuoles were observed in the cytoplasm at all studied times. Although compound **1** caused the aforementioned derangements, the cell membranes as well as the nucleus and the flagellar pocket remained unharmed.

### Immunomodulatory response in macrophages

#### Nitric oxide

To investigate the potential immunomodulatory effect of compound **1** in the host cells, the nitric oxide (NO) content and cytokine levels in macrophages were evaluated after incubation. The results obtained indicated that even after 48 h of treatment with compound **1** (7.5 to 60 µM), the NO levels remained similar to those found in untreated parasites (data not shown). The positive control, LPS (25 µg/mL), was used to achieve increased NO levels.

#### Cytokines

**Fig 10:**
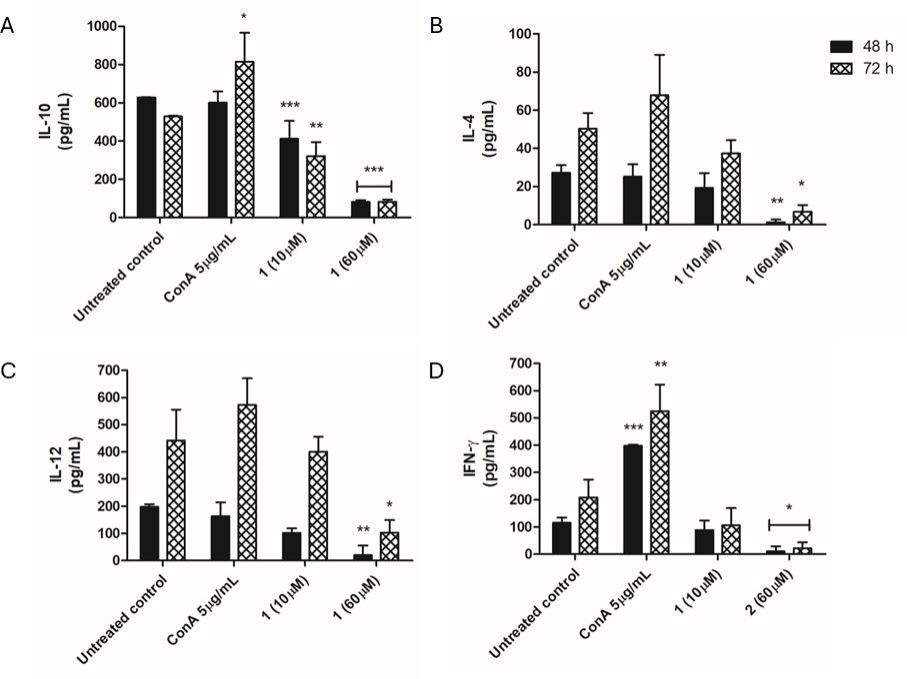
Evaluation of cytokine levels in peritoneal macrophages infected with *L*. (*L.*) *infantum* promastigotes, after treatment with compound **1** (10 and 60 µM) for 48 and 72 h, monitored in a spectrophotometer using the BD OptEIA Mouse ELISA kits (absorbance 450 nm). Untreated and concanavalin A (5 µg/mL) treated macrophages were used as negative and positive controls, respectively. (**A**) IL-10, (**B**) IL-4, (**C**) IL-12, and (**D**) INF-γ. ***P < 0.0002, **0.0025, and *0.05 in relation to the control.

The results obtained show that compound **1** induced a reduction in IL-10 levels after 48 and 72 h of treatment (at 10 and 60 µM), with statistically significant differences to the untreated control (Fig. 10A). Regarding the cytokines IL-4, IL-12, and INF-γ (Fig. 10B through D) significantly reduced levels were observed only at 60 µM, quantified at both treatment times. Therefore, the compound stimulated a reduction in the levels of all studied cytokines at 48 and 72 h. In contrast, the positive control, concanavalin A (5 µg/mL), showed a biological tendency to increase the production of these cytokines.

### *In vivo* pharmacokinetics

**Fig 11:**
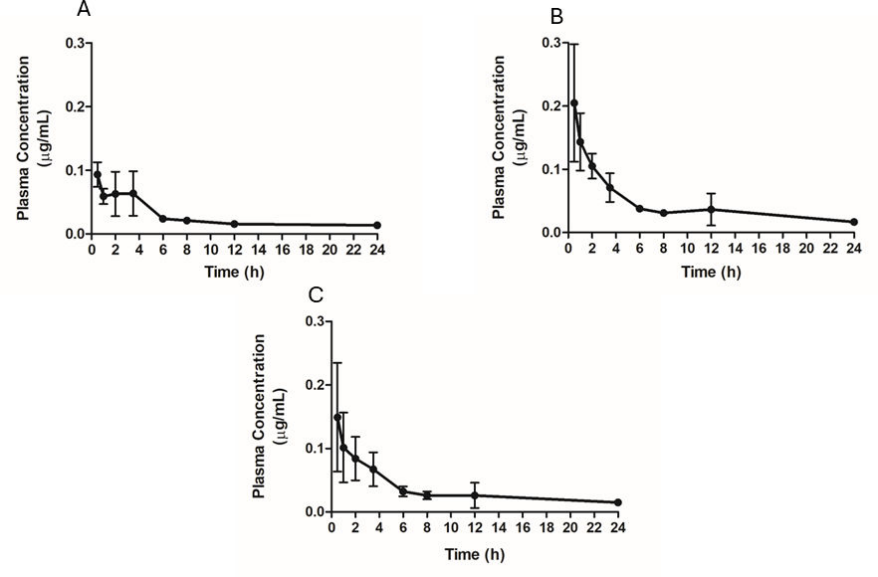
Mean plasma concentration profiles by time, after intraperitoneal administration of compound **1** (5 mg/kg) in six Wistar rats [3 females (A) and 3 males (B)]. (C) A+B. Each point is represented by the mean ± standard deviation.

To investigate the PK profile in an animal model, compound **1** was administered to rats, and the plasma concentration was evaluated by chromatographic techniques. The mean plasma concentration profiles by the time of six rats, previously treated with a single dose of compound **1** (5 mg/kg), are shown in Fig. 11. The corresponding pharmacokinetic parameters are compiled in Table 4. Considering the half-life time results (T_1/2_), it was possible to verify that in male rats, the compound had a slower elimination (31.43 h) than that observed for females (10.72 h). These data were confirmed by the MRT values, which showed intense differences between males (41.08 h) and females (12.75 h). The values of C_max_ and T_max_ also showed significant differences; females resulted in higher plasma concentrations of compound **1** (0.20 μg/mL) than male rats (0.10 µg/mL) in a shorter time (0.50 h versus 1.50 h). The systemic exposure given by the AUC_0-t_ was also lower for males (0.65 µg/mL.h versus 0.98 µg/mL.h).

**TABLE 4:** Pharmacokinetic parameters after intraperitoneal administration of compound **1** (5 mg/kg) in six Wistar rats (3 males and 3 females)

Parameter	Males + females (mean ± SD)
T_1/2_ (h)	21.08 ± 17.72
T_max_ (h)	1.00 ± 1.22
C_max_ (μg/mL)	0.15 ± 0.08
AUC_0-t_ (μg/mL.h)	0.81 ± 0.30
AUC_0-inf_ (μg/mL.h)	1.27 ± 0.34
AUC_extrap_ (%)	0.66 ± 0.22
MRT (h)	26.92 ± 23.18

## DISCUSSION

In the search for new drug leads for visceral leishmaniasis, our group reported the anti-*L*. (*L*.) *donovani* activity of a small family of neolignans isolated from the Brazilian plant *N. leucantha* (Lauraceae) (10). Among them, dehydrodieugenol B was one of the most selective and, in a subsequent analysis, demonstrated promising potential as a prototype for new derivatives with anti-*L*. (*L*.) *infantum* activity (11, 12). Aiming to facilitate structural modifications, a total synthesis of this neolignan was developed, making it possible to perform SAR studies (12, 13).

In addition to the previously reported activity against intracellular amastigotes (IC_50_) and mammalian cytotoxicity (CC_50_), in this work, we also performed a complementary cytotoxicity study prior to the *in vivo* pharmacokinetic profile of one bioactive derivative, compound **1**. Then, the hemolytic activity of this compound was evaluated in BALB/c mice erythrocytes. The results demonstrated that this synthetic derivative induced no hemolysis to the highest tested concentration, similar to the natural product (dehydrodieugenol B) and other chemically related derivatives (11).

Studies of MoA are fundamental during drug discovery and development, enabling the connection of specific molecular interactions to biological activity, and providing important information for molecular optimization (7). To investigate the MoA of compound **1** in *L*. (*L*.) *infantum,* we performed different strategies using fluorescent/luminescent-based approaches by spectrofluorometry, flow cytometry, and also mass spectrometry, and transmission electron microscopy. The plasma membrane is essential for cell survival, being responsible for transporting nutrients and other ions between the intra and extracellular environments. In Trypanosomatids it is constitutively different from the mammalian host and is thus an important target for study (31). In the present work, as a first strategy, the action of compound **1** on the plasma membrane of *L*. (*L*.) *infantum* promastigotes was evaluated using the probe SYTOX Green. After a short incubation time, no alterations in the plasma membrane permeability were observed. Similar results with dehydrodieugenol B derivatives were previously reported in *Trypanosoma cruzi* and *L*. (*L*.) *infantum* (11, 32).

Mitochondria are responsible for numerous metabolic processes, being vital for cell growth and differentiation. In addition, this organelle has been widely studied in Trypanosomatids, as they present a single mitochondrion, unlike mammals that have thousands in their cells. In this context, the search for compounds that affect this organelle has been considered an important approach (33). In particular, neolignans derived from dehydrodieugenol B and eupomathenoid-5 are known to induce mitochondrial membrane depolarization of *Leishmania* and *T. cruzi* parasites (32, 34). In our study, compound **1** caused a significant depolarization of the mitochondrial membrane potential after incubation with *L*. (*L*.) *infantum* promastigotes. Damage in the mitochondrion at early times of incubation can initiate deleterious effects in the bioenergetic system of the cell.

The electrical potential is the result of an electrochemical gradient generated by the respiratory chain and is fundamental in the process of ATP production. Variations in this potential can increase the proton’s permeability in the mitochondrial membrane, leading to failures in oxidative phosphorylation and consequent damage to metabolism, energy deficiency, and parasite death (35). In our study, after 2 h of treatment with compound **1,** alterations in the bioenergetics system of *L*. (*L*.) *infantum* promastigotes were already noticed, with a significant reduction of the ATP levels in the parasites.

The mitochondria are the major source of ROS during the production of ATP. During the respiratory chain functioning, under normal conditions, superoxide anions, hydrogen peroxide, and hydroxyl radicals are generated (36). Therefore, mitochondrial alterations directly affect the production of these species, which when in excess can induce oxidative stress and interfere with metabolic pathways, causing irreversible cellular damage (37). The present study demonstrated a decrease in ROS levels of *L. (L.) infantum* promastigotes after treatment with compound **1**. This may suggest that the mitochondrial failures caused by this neolignan derivative resulted in a respiratory chain collapse, leading to a decrease in cellular energy production (ATP) and a consequent reduction of ROS levels.

Calcium ions (Ca^2+^) play an important role in cell signaling, being essential for the modulation of multiple metabolic pathways and key enzymes (38). Excessive entry through the plasma membrane or failure in any of the regulatory mechanisms leads to an imbalance in cytosolic Ca^2+^ concentrations, making the mitochondria immediately act to control Ca^2+^ levels. However, its capacity for Ca^2+^ uptake is limited, and pronounced variations can lead to the formation of high conductance channels, resulting in dissipation of the mitochondrial membrane potential (39). In our studies, to determine if the mitochondrial imbalance could be ascribed to an indirect effect, the Ca^2+^ levels were investigated in *L*. (*L*.) *infantum* promastigotes after treatment with compound **1**. Our data revealed that after 20 min of incubation, the compound promoted a rapid increase in Ca^2+^ levels of the parasite. Therefore, it can be suggested that the mitochondrial dysfunction of *Leishmania*, observed in the presence of compound **1,** may be ascribed to Ca^2+^ imbalance. However, a direct action of compound **1** in the mitochondria cannot be totally excluded. Due to the elevated Ca^2+^ levels in *Leishmania*, we investigated the potential participation of the acidocalcisomes. These organelles are the main Ca^2+^ reservoirs in trypanosomatids, unlike mammalian cells where the endoplasmic reticulum performs this function (38). Acidocalcisomes exhibit an acidic pH that favors Ca^2+^ retention since Ca^2+^/H^+^ transporters are responsible for the efflux of this ion. Therefore, if an alkalinization occurs, these organelles internalize H^+^, releasing Ca^2+^ into the cytosol to reestablish the homeostatic balance (40). In our studies, after a short incubation (20 min) with *Leishmania*, compound **1** induced a rapid alkalinization of the acidocalcisomes. Thus, it is possible to infer that this neolignan disturbs the function of these organelles, triggering the release of Ca^2+^ in the cytosol. Miltefosine, the only oral drug available for the treatment of leishmaniasis, presents a similar mechanism in *L*. (*L*.) *donovani*, causing alkalinization of acidocalcisomes with a subsequent increase in Ca^2+^, leading to the collapse of the mitochondrial membrane potential (41).

From the catalysis of chemical reactions to the growth and adaptation to different microenvironments, proteins are essential for the execution and regulation of several cellular mechanisms. Due to its important role in parasite viability and infectivity, protein imbalance can lead to considerable dysfunction in the cell metabolism, leading to death (42). In our studies, it was possible to observe that compound **1** induced an increased level of total protein of *L*. (*L*.) *infantum* promastigotes. This effect can possibly be attributed to a regulatory mechanism for cell survival in the face of an eventual metabolic collapse, caused by bioenergetic breakdown in the parasite.

The cell cycle is strictly controlled by a complex of proteins, whose role is essential for parasite survival. Compounds that target these proteins or cause some dysfunction in cell division can generate serious proliferation disorders, leading to parasite death (43). Additionally, our previous work revealed that a dehydrodieugenol B derivative interfered with the cell division of *L*. (*L*.) *infantum* parasites, compromising DNA replication and mitosis (11). Here, modifications in the cell cycle of *L*. (*L*.) *infantum* promastigotes were evaluated by flow cytometry, which showed that compound **1** interferes in the SubG_0_ and G_0_/G_1_ phases. The increase in cells in the SubG_0_ phase indicates that the DNA content is fragmented, while the decrease in G_0_/G_1_ indicates a smaller number of parasites in the stationary state, preparing for the division. Together, these data may suggest that the increase in SubG_0_ occurs at the expense of cells in the G_0_/G_1_ phase, without significant changes in the mechanism of DNA replication and mitosis (phases S and G_2_/M).

Programmed cell death (apoptosis) is evidenced by several events, such as depolarization of the mitochondrial membrane, increased levels of ROS, and exacerbation of cytosolic Ca^2+^. Furthermore, fragmentation of the internucleosomal genetic material is considered an extremely important indicator in this type of cell death (44). Considering the results obtained in the mitochondrial and cell cycle studies, a fragmentation analysis of the genetic material was carried out in *L*. (*L*.) *infantum* promastigotes treated with compound **1**. Using electrophoresis in agarose gel, it was possible to verify that this neolignan derivative induced no alterations in the integrity of the genetic material. Thus, it is possible to infer that the DNA fragmentation cannot be attributed to a mechanism of action of compound **1**. Additionally, based on the cell cycle study, we can suggest that the increased cells with fragmented DNA (subG_0_ phase) may not be ascribed to an “apoptosis-like” death.

Considering that necrosis involves the rupture of the plasmatic membrane, this type of cell death can also be discarded since compound **1** does not induce the formation of pores in this membrane. Consequently, the hypothesis of autophagy may be considered, which is a process characterized by the degradation of damaged cell constituents, forming multivesicular bodies and vacuoles in the cytoplasm (45). Through an ultrastructural investigation using transmission electronic microscopy, it was possible to verify the vacuolation in the cytoplasm of *L*. (*L*.) *infantum* promastigotes after treatment with compound **1** at all times of incubation. Additionally, mitochondrial disorders were also verified, corroborating the studies carried out by flow cytometry. No alterations in the plasmatic membrane and nucleus were observed, reinforcing the hypothesis that the compound does not induce cell death by apoptosis or necrosis. Hence, the intense cellular stress caused by this neolignan possibly causes an autophagic cell death.

In a complementary manner, chemotherapeutics can also induce immunomodulation of host cells, increasing the production of molecules responsible for defense against intracellular pathogens such as cytokines, NO, and ROS. Thus, in addition to the direct action on the parasite, drugs can rely on the activation of host cells to eliminate the infection (46). Considering the selectivity of compound **1** against intracellular amastigotes of *L*. (*L*.) *infantum*, the influence of this neolignan on immunomodulatory mechanisms was studied through the measurement of nitric oxide and cytokines.

NO promotes the control of *Leishmania* infection by decreasing the metabolic activity of intracellular amastigotes, restricting the growth, and survival (46). The present data demonstrated that compound **1** induced no significant alterations in the NO levels of macrophages, suggesting a lethal effect via a NO-independent course.

Cytokines play a key role in determining resistance or susceptibility to leishmaniasis, being present throughout the infection. Adequate control of the disease requires a balance between the Th1 and Th2 response, as the predominance of a Th1 response can lead to tissue damage, while the predominance of a Th2 response can result in the progression and exacerbation of the disease (47). Previous studies with the natural product dehydrodieugenol B demonstrated the reduction of IL-6 and IL-10 cytokines (10). In the present work, treatment of *Leishmania*-infected macrophages with compound **1** resulted in the reduction of Th1-response cytokines (IL-12 and INF-γ) and Th2-response cytokines (IL-4, IL-10). As such, it is possible to suggest that compound **1** also exerts an immunomodulatory effect on the host cells, characterized as an anti-inflammatory response, which may be beneficial to the outcome of the disease.

According to Tuntland et al., the implementation of pharmacokinetic and pharmacodynamic strategies in early research phases of drug discovery is now a consensus in the pharmaceutical industry and must precede *in vivo* efficacy studies (48). Pharmacokinetics is characterized by the study of drug behavior in the body, incorporating processes of absorption, distribution, metabolism, and excretion. Therefore, its investigation in the initial stages of the drug pipeline can contribute to the design of a rational *in vivo* efficacy study (49). In this context, aiming toward future pre-clinical studies of efficacy in animal VL-models, a pharmacokinetic profile investigation of compound **1** was carried out in Wistar rats after intraperitoneal administration of a single dose (5 mg/kg).

It is also recommended that plasma half-lives should not be elevated, inducing parasitic resistance. Miltefosine, the only oral drug available for the treatment of leishmaniasis, presents a long T_1/2_ of approximately 126 h, and parasitic resistance has been described (50). Our data revealed that the systemic exposure and the maximum plasma concentration values were below our IC_50_ values obtained in intracellular amastigotes. Studies with repeated doses, different doses, and different routes of administration should be adequate to estimate an adequate administration regimen and prevent possible therapeutic failures. The concentration and maximum time results reaffirm the disparity in the pharmacokinetic responses of the different sexes, with females presenting twice the plasmatic concentration of compound **1** in a shorter time. Intrinsic factors such as sex, weight, metabolic disorders, and genetic polymorphisms can directly affect the systemic exposure of a compound in the body, which may generate divergent results between individuals (51). It is possible to verify that in both sexes, compound **1** reaches its maximum concentration in a short time, but remains in the organism long enough to suggest a daily dosage interval may be possible to promote its therapeutic action.

### Conclusion

In summary, the investigation of the mechanism of action of compound **1**, a semisynthetic derivative of natural product dehydrodieugenol B, showed a pronounced effect on acidocalcisomes with concomitant intracellular Ca^2+^ extravasation. Together, these effects induced mitochondrial impairment and subsequent collapse of the bioenergetic chain, decreasing ATP and ROS levels. It was also observed that compound **1** had no direct effect on the replication mechanism of the parasite, causing no damage to the integrity of the genetic material. At an ultrastructural level, the formation of cytoplasmic vacuoles was observed, suggesting an autophagic cell death for Leishmania. Additionally, compound **1** showed a NO-independent leishmanicidal action, with an immunomodulatory effect characterized by an anti-inflammatory response. Finally, the pharmacokinetic profile in rats after a single dose, demonstrated adequate half-life and other parameters to support future *in vivo* efficacy studies in VL-models. Therefore, in this work, we propose compound **1** as a promising candidate for future preclinical studies.
